# Enhancing Physical Activity and Brain Reorganization after Stroke

**DOI:** 10.1155/2011/515938

**Published:** 2011-07-03

**Authors:** Janet H. Carr, Roberta B. Shepherd

**Affiliations:** Faculty of Health Sciences, The University of Sydney, P.O. Box 170, Lidcombe, Sydney, NSW 1825, Australia

## Abstract

It is becoming increasingly clear that, if reorganization of brain function is to be optimal after stroke, there needs to be a reorganisation of the methods used in physical rehabilitation and the time spent in specific task practice, strength and endurance training, and aerobic exercise. Frequency and intensity of rehabilitation need to be increased so that patients can gain the energy levels and vigour necessary for participation in physical activity both during rehabilitation and after discharge. It is evident that many patients are discharged from inpatient rehabilitation severely deconditioned, meaning that their energy levels are too low for active participation in daily life. Physicians, therapists, and nursing staff responsible for rehabilitation practice should address this issue not only during inpatient rehabilitation but also after discharge by promoting and supporting community-based exercise opportunities. During inpatient rehabilitation, group sessions should be frequent and need to include specific aerobic training. Physiotherapy must take advantage of the training aids available, including exercise equipment such as treadmills, and of new developments in computerised feedback systems, robotics, and electromechanical trainers. For illustrative purposes, this paper focuses on the role of physiotherapists, but the necessary changes in practice and in attitude will require cooperation from many others.

## 1. Introduction

It is well established that stroke survivors have low levels of physical fitness and muscle strength that impact on their ability to perform everyday activities and affect their independence and community participation. Physical inactivity after stroke has many negative sequelae that may impact upon potential reorganization of brain function and on recovery of motor abilities. There is greater awareness now of the need for patients to practice everyday actions intensively in order to regain skill in motor performance. However, it is only recently that interest has turned to the physiological effects of inactivity in bringing about a reduction in aerobic capacity and physical endurance and to the effect of this deconditioning on an individual's capacity to raise the energy levels in motor training that are necessary to improve functional performance and brain reorganization. Physical activity is positively related to aerobic capacity, the capacity of the cardiorespiratory system to supply oxygen (the cardiac output) and of skeletal muscle to utilize oxygen.

Low aerobic capacity limits performance of activities of daily life and increases the risk of falls and dependence on others. It is also a significant determinant of poor bone health (specifically osteoporosis) in individuals with chronic stroke [1]. Reduced cardiorespiratory fitness and inactivity after stroke appear to be related to a combination of pathological (comorbid cardiovascular disease, coronary artery disease), physiological (decreased muscle activation and motor control, and changes in muscle length and stiffness), and environmental factors (little opportunity or incentive for physical activity). For the elderly, these factors may be combined with an age-related decline in cardiorespiratory fitness reported to be approximately 10% or greater per decade [2]. 

Environmental factors contributing to declining fitness during rehabilitation may include excessive bed rest and physical and mental inactivity. Observational studies in the USA and Europe, carried out in the 1980s, found that the majority of a patient's day in hospital rehabilitation may be spent being alone and inactive [3, 4]. A similar situation was observed more recently in an Australian institution where only a small proportion of the day was spent in physical activity [5, 6]. One study of physical activity within the first 14 days of acute stroke found that although most patients, 84.5%, were free to move out of bed, more than 50% of the patients' time was actually spent resting in bed. Only 13% of individuals observed were engaged in activities that might have the potential to improve mobility and prevent secondary complications [7]. A multicentre longitudinal project has compared inpatient care and recovery patterns between four European rehabilitation centres. Patients in the UK centre spent less time in therapy and more time sitting inactive by their beds than those in the German or Swiss centres, with UK therapists spending more time doing legally required administrative tasks with less time to care for their patients (1 hour per day) [8, 9]. 

Clearly, if meaningful and challenging physical and mental activity play a critical part in driving optimal brain reorganization, there is an urgent need throughout the world for patients to be more active during their hospital rehabilitation than they currently tend to be. However, it is not only the time spent in active exercise that is significant but whether what the individual is doing in that time is sufficiently vigorous and relevant to induce a training effect not only on motor skill but also on level of fitness. A study of patients in physiotherapy and occupational therapy between 2 and 14 weeks after stroke, during which heart rate was monitored, indicated that therapy may be of insufficient intensity to produce a cardiorespiratory training effect [10]. On average, in a physiotherapy session, 42% of the time was spent inactive in lying, 11% active in lying, 16% active in sitting, and 31% active in standing. If it was present, the aerobic component of a typical physiotherapy session lasted less than 3 minutes. Although one might expect progressively higher exercise intensities over time as functional status improved, any increase in HR_ mean _ and HR_peak_ did not reach statistical significance. During occupational therapy a considerable percentage of time was spent in sitting, discussing issues related to discharge, equipment needs and home management. 

The reorganization of rehabilitation to enable specific task training and practice, with the progressively increasing intensity of exercise required not only for motor learning but also directed specifically at muscle strength and endurance, and aerobic cardiorespiratory fitness, is a major challenge. In this paper we argue for increased effort to be made to reorganize therapy delivery after stroke to take account of these needs, in particular specific cardiorespiratory training, and we give some examples of how this might be achieved [11, 12].

## 2. Early Mobilization

It is well known that people respond negatively to prolonged physical inactivity. However, after stroke the consequences may be particularly severe, since the reorganization of the brain that follows the neural lesion is thought to be dependent on what the patient does and the meaningfulness of this, as well as the environment in which activity takes place. Early onset of active and moderately vigorous rehabilitation, especially of weight-bearing activities, could be expected to influence not only the rate and extent of physical recovery, by preventing neuromuscular disuse changes related to long periods of rest, but also to have a role in minimizing cognitive deterioration, depression, and anxiety. 

Early mobilization (helping patients to get out of bed into sitting, standing up, and walking) is now recommended in a number of clinical guidelines for acute stroke (e.g., [13]). In general, if there is no progression of neurological deficits, active rehabilitation should commence within 24–48 hours [14]. The few existing studies provide limited but promising evidence of the benefits of early exercise on functional recovery, as well as on early depression [15], medical complications [16], health care costs [17], and harm minimization. A recent Cochrane review concluded that although further research is needed in this field, the evidence suggests that stroke units practicing early mobilization as part of standard care should continue to do so [16].

There is evidence that early, frequent, and active mobilization can influence recovery. A stroke unit in Trondheim, Norway, has been practicing early active mobilization for 20 years. A physiotherapist and nurse take a staged approach, first sitting the patient up in bed, and then over the edge of the bed, then into a chair or into standing as appropriate. Once this first mobilization has been successfully completed, the patient is then assisted frequently throughout the day to repeat out of bed activities, progressively increasing activity as able [18]. A study in 2008 compared physical activity levels of rehabilitation patients in this early mobilization centre with stroke units in Melbourne, Australia [19]. Patients had similar baseline characteristics. It was found that Trondheim patients spent twice as long standing and walking and less time lying in bed. Patients were observed to change position from lying to sitting to standing twice as often as patients in Melbourne. 

These results are only meaningful if it can be shown that early physical activity actually does improve patient outcome, and there is some indirect evidence that it has a positive effect [14]. It is also apparent that greater intensity and frequency of therapy can improve outcome [20]. A large, international multicentre randomised controlled trial, the Australian Very Early Rehabilitation Trial (AVERT), is currently underway [21] aimed at testing whether early and frequent mobilization results in fewer deaths and less disability at 3 months after stroke compared with current stroke practice. Results are expected in 2012. 

The recognition of the specificity of neuromuscular processes and resultant development of a task-specific training and practice model of rehabilitation with emphasis on activating motor learning processes, provides a framework for functional rehabilitation, and biomechanics and exercise physiology provide the means to combine skill and aerobic training methods even in this early phase of rehabilitation [11, 12]. For example, simple methods of providing increased intensity in the practice of significant actions in this early period include specific and repetitive practice of standing up and sitting down, aimed both at improving the effectiveness of performance of standing up itself but also the person's ability to perform the action with sufficient repetitions to produce an efficient performance in terms of O_2_ uptake. The ability to stand up and sit down is critical to the rehabilitation process, and biomechanical principles provide guidelines to reducing the effort required to stand up in the very early stages of mobilization [12]. For example, biomechanical investigations have shown that positioning the feet back to 75° ankle dorsiflexion and raising the seat height each reduces the muscle forces required.

Walking on a treadmill with body weight support via harness connected to an overhead support system may be the only means of practicing walking for more severely affected weak and dependent individuals early after their stroke, and it is reported to be well tolerated and effective [22]. Without the treadmill and harness, practice of walking will be delayed. Treadmill walking also allows for independent and semi-supervised practice for those with more ability [23], as well as improving aerobic capacity [24], and increasing walking speed and endurance [25]. The very early provision of assisted overground and harness-supported treadmill walking is probably critical to good postdischarge functional capacity in terms of both performance and energy levels.

An early goal for walking overground and independently is to walk to the next appointment or to walk at least part of the way rather than being transported in a wheelchair. Each day the patient selects a distance to walk independently and safely. It may initially be a few steps. The goal can be to walk this distance 5 times a day, increasing distance as soon as possible, and keeping a record of progress which gives the patient a specific attentional focus. The results of several motor learning studies in which the person's attention was focused on the outcome of an action rather than the action itself (or the “quality” of the movement), have shown that, for example, walking to a prearranged place as quickly as possible can produce more effective performance than focusing attention on the quality of the movement [26]. In early practice of overground walking, a sense of security is provided if the therapist or nurse assists by holding a belt with grab handles (e.g., Handi-lift/Walk Belt, Pelican Manufacturing, Osborne Park, Western Australia 6017).

## 3. Exercising during Rehabilitation

It is increasingly acknowledged that the rehabilitation team must adapt the delivery of interventions, increase the time spent in meaningful exercises and task practice, to meet the needs of brain reorganization, skill relearning, and improved physical and mental fitness. The low intensity, low duration, and low frequency of physiotherapy common in many rehabilitation centres, following outdated and insufficiently challenging therapy models of intervention, sometimes for as little as 30 minutes to one hour per day, severely restrict the optimal level of recovery required for participation in daily and social activities after stroke. 

Therapists need to move away from reliance on one-to-one therapy to a model in which the patient practices not only in individualized training sessions with a therapist but also in groups, and in circuit training, where patients practice at work stations set up for weight-bearing strength training exercises, and to encourage practice of specific actions. Patients are semisupervised and assisted as necessary by therapist and aide, with similar attitudes and methods to those who work in sports training. 

The modern rehabilitation workspace needs to provide an environment built to encourage and challenge physical activity. Such an environment includes a suspended harness system that allows practice of balancing and walking tasks, feedback and computerized devices to provide information and incentive, exercise machines such as treadmills with harness suspension, stationary bicycles, including an electronically braked isokinetic ergometer [11], and stepping machines. Some centres are developing and testing electromechanical training aides [27], including robotic devices [28] and virtual reality systems [29]. Even simple technological aids can increase time spent in physical and mental activity by enabling patients to practice independently [30]. Assistive devices and the assistance of therapy aides can drive physical participation and increase intensity of practice, motivation, competition, and personal responsibility. Patients can also work in a twosome, one assisting the practice of the other [31].

For both therapist and patient, the focus of physiotherapy is always on the optimization of functional motor actions critical to everyday life. Optimization in this context refers to training a person's performance to be as *efficient* and as *effective* as possible in achieving a functional goal, “effective” referring to a goal achieved and “efficient” meaning at the least physiological cost. For this latter, the focus is particularly on exercising the large muscles of the lower limbs and trunk, a requirement of cardiorespiratory fitness training [32]. Mobility requires lower limb muscles that are strong enough and well enough coordinated to support, transport, and balance the body mass in a gravitational environment, with muscles capable of producing force quickly enough and with the appropriate timing (i.e., power) to avoid unexpected trips and slips. All of this requires appropriate energy levels and aerobic fitness.

As an example, take an action that is performed many times in daily life. The ability to stand up and sit down effectively without falling is essential to independent living as well as a critical prerequisite for upright mobility, yet it is one of the most physically demanding actions we perform regularly, requiring greater strength of lower limb extensors and range of motion of hips, knees, and ankles than walking or stair climbing [33]. Lack of independence in this action is a common source of falls [34, 35], and is one of the most likely factors associated with institutionalization [36].

 Guidelines for training: standing up and sitting down based on biomechanical experimentation and clinical evidence, are provided in a recent textbook [12]. In a randomized controlled study of a program for training standing up after stroke, Britton and colleagues assessed the effects of a 30 min standardized training program of standing up and sitting down [37]. The aim of each session was to increase the number of repetitions, and the number of stands and sits was recorded throughout each day using an ActivPal accelerometer activity monitor. The number of repetitions in the exercise group increased on average from as few as 18 per day to 50 stands. In addition, in this group there was a significant mean increase in percentage of body weight taken through the paretic (10%) leg after 1 week of training. In contrast, in the control group, the weight taken on the paretic leg decreased during the period of the clinical trial. The strength of this study is in the prescribed protocol which included not only the number of repetitions per day but also a record of what the patient actually did and how many repetitions, that is, the dosage. This latter is notable, as “dosage” of therapy has not been an issue in rehabilitation until recently and has rarely been reported in clinical trials. 

The ability to regain functional motor skills and increase the intensity of exercise and practice in rehabilitation involves in large part the acquisition of balance [12]. Central to balance control is the need to keep the centre of mass within the perimeter of the base of support, or on track to a new base of support as in walking or running [38]. It is becoming increasingly clear that although there are many different types of exercise available, some are more likely to improve balance and prevent falls than others, but overall it appears that balancing mechanisms are very specific to the action being performed, the purpose of the action (the task) and the environment in which it takes place. There is increasing evidence that challenging balance exercises in standing with the feet close together and exercises for the lower limb muscles performed in standing against body weight resistance [39, 40], for example, in standing up and sitting down, step ups, heels raise and lower, marching, stair walking, semisquats, and reaching to the floor sideways and forward to pick up an object, performed with increasing numbers of repetitions and without reliance on the upper limbs for support and balance [12] are the optimal way to improve balance as well as flexibility, strength and endurance, and fitness. These exercises can be made more challenging by increasing the height of steps and chairs and by increasing and varying speed [41].

Despite the positive changes taking place in physical rehabilitation, it appears to be common for deconditioning to be evident in the stroke community at discharge from rehabilitation. We know that physical activity is positively related to aerobic capacity [42] and improving aerobic capacity during rehabilitation must be addressed if individuals are to become part of their community again. The American College of Sports Medicine provides guidelines for aerobic training [43]. Further information on testing aerobic fitness, methods of increasing intensity of effort as a person improves, and the monitoring of physiological responses is provided in several publications (see [11, 42, 44]).

The rehabilitation period, often very short, provides an opportunity to ensure that patients are not only prepared for discharge but also understand the need to continue with physical activity after discharge in order to increase their motor skill and fitness levels.

## 4. Continuing Exercise and Increasing Physical Activity after Discharge

The promotion of a physically active lifestyle has become an important issue in health policy in many countries. The World Health Organization (WHO) has recognised this by modifying and revising the classification of impairments, disabilities, and handicaps. The most recent International Classification of Function (ICF) provides greater focus on quality of life and integration of physical activity into daily life, emphasising “limitation in activity” rather than disability and “restriction in participation” rather than handicap [45]. 

Recently, WHO issued a warning that a sedentary lifestyle is one of the most serious, yet insufficiently, addressed public health problems of our time, and this is of course as relevant to people disabled through stroke as it is to anyone else. Although many people affected by stroke regain the ability to walk by the time they are discharged from rehabilitation, it is well documented that many have sufficiently low physical endurance to limit their ability to perform household tasks or even to walk short distances. Individuals who are discharged showing improvements in gait are not necessarily functional walkers. It has been found that walking may require a much higher level of energy expenditure than before the stroke [46], and that this can limit the ability to walk outside the house. Minimum criteria for successful community walking include an independent walking velocity of 0.8 m/s or greater, the ability to negotiate uneven terrain and kerbs, and the physical endurance to walk 500 m or more. In a review of 109 people discharged from physiotherapy, only 7% achieved the minimum level [47]. Cardiorespiratory fitness training can address both the efficiency with which people affected by stroke can walk and the distance they are able to achieve [11].

The loss of independent ambulation outdoors has been identified as one of the most debilitating of stroke sequelae. Among stroke survivors one year after stroke, the most striking area of difficulty was low endurance measured by the distance walked in a 6-minute walk test (6MWT) [48]. Those subjects able to complete this test were able to walk on average only 250 m compared to the age-predicted distance of >600 m, equivalent to 40% of their predicted ability and not far enough for a reasonable and active lifestyle. The detrimental effect of low exercise capacity and muscle endurance on functional mobility and on resistance to fatigue is likely to increase after discharge if follow-up physical activity and exercise programs are not available. 

A physically active lifestyle has several fitness and health benefits and is also critical for stroke survivors. Many levels of functioning can be influenced positively. Exercise helps maintain fitness and participation, but also has a very real protective function, helping to prevent recurrent stroke and further cardiovascular disease, reducing the risk of falls, increasing physical independence and improving quality of life [49]. In addition, a randomized controlled trial provides evidence of increased activity of cortico-subcortical networks produced by repetitive treadmill training of stroke-affected individuals. Similar changes were not found in the control group who had a comparable program that provided neither aerobic stress nor required many active repetitions [50]. Exercises directed at maintaining or improving aerobic function, strength and endurance, flexibility, and neuromuscular control are all strongly recommended for stroke survivors in order to further the recovery process, increase confidence and improve well-being [49]. 

Currently, the challenge for many survivors is to find in their local community both a person with the expertise to assist with the prescription and supervision of their program of exercise and aerobic training, and the location of a suitable community-based venue. Physiotherapists and exercise science graduates are well placed to play a role in the development of poststroke community-based exercise programs that include aerobic exercise and provide additional skill training, as well as offering help and advice about ways of increasing physical activity throughout the day.

The major forms of exercise to improve physical activity for individuals after stroke include, as well as aerobic fitness training [11, 42, 44], functional task and balance training [1, 41, 51–56], and strength and endurance training [41, 55, 56]. A program can include, in addition to task-relevant exercises, a combination of aerobic, strengthening, and flexibility exercises (see [57]). Active exercise can enhance physical activity and exercise tolerance if well prescribed, but exercises need to be sufficiently intensive and in weight-bearing positions. Exercises that share similar biomechanical characteristics, for example, exercises that involve flexion and extension of hips, knees and ankles over the feet as a fixed base of support, are likely to enable a transfer of strength gains to improved stair walking, squatting, and standing up and sitting down [12]. Exercises in water may also have positive training effects, and when properly supervised can increase fitness. They may be particularly useful for people with painful arthritic joints who find it difficult to exercise [58].

An interesting outcome of organized postdischarge exercise programs for chronic stroke patients has been the collegiality that develops among participants and the encouragement and support they provide to each other [53]. Similar observations regarding the social benefits of group community exercise programs have been reported by others [55, 59]. Increased self-esteem and a feeling of well-being and of being in control that is engendered by an exercise program can give individuals the confidence and drive to improve their performance in other activities. On completion of an exercise program on stationery bicycles, for example, participants reported increased self-esteem and several reported changes in their general behaviour—one participant started gardening again and another could once again comb her hair [60]. Time since stroke does not appear to have a limiting effect on brain plasticity [61]. We know from findings of recent studies that it is possible, even several years after stroke, for people to make positive gains in strength, mobility, fitness, endurance, and skill in semisupervised community classes [51–56]. Several of these programs had an impact on activities such as getting on and off a bus and socializing with friends away from their homes, all activities considered meaningful to the individuals. Interviews with participants and their families suggested that quality of life gains are critical factors in reintegrating stroke survivors into society. 

Similarly, aerobic capacity improves with appropriate training even a long time after stroke [11, 42]. Methods used in different trials have included cycle ergometry [62], treadmill walking [63, 64], and a combination of aerobic and strengthening exercises [54, 56]. Although the effects are exercise-specific, generalization occurred in the improvements noted in general health and well-being that positively affected those activities the participants considered meaningful. A recent systematic review provides good evidence that aerobic exercise, at 50–80% heart rate reserve, on 3–5 days a week for 20–40 minutes should be an important component of stroke rehabilitation and postdischarge training [42].

Motivation to start exercising after discharge depends on several factors—availability of community-based group exercise programs, previous exercise experience, knowledge about exercise and general health, and psychological factors [65, 66]. Availability of transport and/or dependency on another can affect participation as does absence of feedback. Feedback in relation to improved balance, distance walked, or increased muscle strength may provide additional incentives for continued participation. Exercise diaries or practice records can provide information to encourage individuals to continue to exercise after input from an instructor. Social support is an important mediator of adherence and since effects of exercise are not always immediately apparent, social support is especially important at the beginning of a program. There is some evidence that most of those who participate in exercise programs for six months are likely to continue to do so.

## 5. Conclusion

Although it is evident that in general more intensive rehabilitation results in better recovery, evidence from studies over a number of years suggests that physical rehabilitation is not intensive or targeted enough to have a training effect or even to prevent secondary adaptive changes in muscle and metabolism that underlie the profound cardiovascular and muscular deconditioning [67]. Several reports indicate low physical endurance and cardiovascular fitness levels on discharge. There is evidence that those who demonstrate improved walking at discharge are not necessarily functional walkers when faced with the energy requirements of real life nor are they confident enough to walk outside the house. It is becoming clear that the process of regaining independence and building up energy levels requires more active and intensive inpatient rehabilitation than commonly available, together with a comprehensive plan for a post-discharge continuation of physical activity and the development of exercise opportunities within the community. The knowledge and evidence are available. It should therefore be an expectation that clinical practitioners will follow specific guidelines based on this knowledge and on the evidence from clinical research, and that they will test functional outcomes of specific interventions at regular intervals using valid and reliable measures. For movement rehabilitation after stroke to achieve the goals expressed here, physiotherapists who have no background in exercise science or biomechanics need to upgrade their knowledge and skills in appropriate educational settings.
